# Severe emphysematous pyelonephritis managed with staged percutaneous drainage – A Changing paradigm

**DOI:** 10.1016/j.eucr.2025.103033

**Published:** 2025-03-31

**Authors:** Shravankrishna Ananthapadmanabhan, Zoe Williams, Ramesh Shanmugasundaram, Henry Wang, Nicholas Mehan

**Affiliations:** Nepean Hospital Urology Group, Nepean Hospital, Kingswood, New South Wales, 2747, Australia

## Abstract

Emphysematous pyelonephritis (EPN) is a potentially life-threatening infection of the renal parenchyma and perinephric tissues by gas-forming organisms. Historically, open surgical drainage or nephrectomy was the standard of care, however, in recent years percutaneous drainage as a minimally invasive approach has become popular. However, whether percutaneous drainage can be successful in more severe cases of EPN is less clear. We report a case of severe Class IIIb EPN managed successfully with multiple staged percutaneous drainage procedures guided by interval re-assessment of clinical, biochemical, and radiological progress in a 52-year-old male with poorly controlled Type 2 Diabetes.

## 1 Introduction

Emphysematous pyelonephritis (EPN) is a life-threatening, necrotising infection of the renal parenchyma and perinephric tissues by gas-forming organisms.^1^ The concept of emphysematous urinary tract infections was introduced in 1898 by Kelly and MacCallum who proposed that fermentation by certain organisms would cause pneumaturia.^2^ The term emphysematous pyelonephritis was subsequently coined in 1962 to describe this process.

Classically, EPN occurs in diabetic or immunocompromised patients, or in the setting of obstructive uropathy, and is more common in female patients.^1^ The commonest pathogens isolated from patients with EPN include Escherichia coli followed by Klebsiella pneumoniae, with other culprit organisms including Proteus and Pseudomonas species, or rarely fungal organisms such as Candida species.^3^ Gas production occurs during the fermentation of glucose into hydrogen and carbon dioxide gas and this process is facilitated in diabetic patients by glycosuria, blunted immune responses, and microangiopathy which promote the propagation of infection. Urinary obstruction produces parenchymal ischaemia and necrosis, favouring infection. In non-diabetic patients with EPN, obstructive uropathy is almost always present.^4^

Imaging is essential to the diagnosis of EPN, with computed tomography (CT) being the gold standard, demonstrating the presence of gas within the renal parenchyma, collecting system, or perinephric tissues. Two subtypes of EPN were identified by Wan et al., in 1996 based on patterns of gas distribution and parenchymal destruction. Type I EPN was characterised by extensive parenchymal destruction with minimal perinephric fluid collections and a streaky or mottled gas pattern, whilst Type II EPN was defined by fluid collections with locules or bubbles of gas.^4^ In 2000, Huang and Tseng developed a radiological classification of EPN based on the extent of gas (Table 1).^4^ These classifications were of prognostic significance, with Type I EPN, and a greater degree of gas extension generally predictive of poorer outcome.^4^^,^^5^ Additionally, imaging is essential in identifying whether upper tract obstruction is present so that urgent decompression can be pursued.

**Table 1:** – Huang and Tseng classification of emphysematous pyelonephritis.

Huang and Tseng Class of Emphysematous Pyelonephritis	Extent of gas
Class I	Gas in the collecting system
Class II	Gas limited to the renal parenchyma
Class IIIb	Gas extending into the perinephric space
Class IIIb	Gas extending into the pararenal space (gas extension beyond Gerota's fascia)
Class IV	Bilateral emphysematous pyelonephritis or EPN in a solitary kidney

Historical approaches to managing EPN involved either medical management alone with intravenous antibiotics in milder cases, or in conjunction with nephrectomy or open surgical drainage for source control. EPN was a fatal disease, with a reported mortality of up to 50 % depending upon patient and disease factors, with poorer outcomes in non-surgically managed cases.^1^ Recently, there has been a paradigm shift towards percutaneous drainage as a minimally-invasive, nephron-sparing alternative to surgery in managing EPN with positive outcomes reported in a number of case reports and case series'.^3^ Subsequent attempts at risk stratification to identify clinical, biochemical, or imaging characteristics that would identify patients suitable for minimally-invasive approaches and those who would require early nephrectomy have been inconclusive.^6^ Whether percutaneous drainage is a feasible technique in patients who have extensive unilateral EPN, or other high-risk features is not established, and a patient-specific approach is needed.

We present a case of severe class IIIb emphysematous pyelonephritis in a 52-year-old male successfully managed with multiple staged percutaneous drain insertions over a period of 32 days. To our knowledge, this is the first reported case of EPN managed using multiple planned percutaneous drainage procedures based on frequent assessment of radiological, clinical, and biochemical progress until resolution. This supports the growing paradigm that EPN can be initially managed using percutaneous drainage, with nephrectomy reserved for cases where patients have clinical deterioration despite minimally-invasive source control.

## 2 Case description

**Fig. 1:**
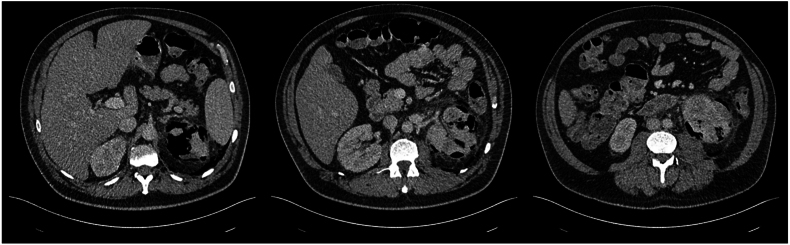
– CT Abdomen and Pelvis (CTAP) on admission demonstrating Class IIIb Emphysematous Pyelonephritis with retroperitoneal and para-renal gas extension.

A 52-year-old male presented to the Emergency Department of a sub-acute hospital with 2 days of lethargy and high-grade fevers. On presentation, he met sepsis criteria with hypotension, tachycardia, and altered mental status, and received 6 L of intravenous crystalloid to maintain a non-invasive mean arterial pressure (MAP) greater than 60 mmHg. The patient reported no focal infective symptoms, though, initial investigations revealed a random blood sugar level of 29.0mmol/L, and a urinalysis positive for leukocytes and nitrites (Table 2). A CT of the Abdomen and Pelvis with portal venous phase contrast revealed severe left-sided Huang and Tseng class IIIb emphysematous pyelonephritis with retroperitoneal gas extension (Fig. 1).

**Table 2:** – Summary of relevant biochemical investigations on admission.

Investigation	Result
pH	7.45
pCO2	26 mmHg
HCO3	18mmol/L
Base excess	−4.5mmol/L
Sodium	123mmol/L
Lactate	2.4mmol/L
Urea	10.0mmol/L
Creatinine	124mmol/L
C reactive protein	342mg/L
White cell count	8.7 x 10^9^ cells/L
Platelets	107 x 10^9^ cells/L
Neutrophils	7.6 x 10^9^ cells/L

The patient was transferred urgently to a tertiary hospital with a Urology service for further management. Initial management involved multidisciplinary discussion with the Infectious Diseases, Intensive Care, Endocrinology, and Radiology departments, and included urethral catheterisation, broad-spectrum antibiotic coverage with Piperacillin-Tazobactam and strict glycaemic control with a basal-bolus insulin regime. Initial imaging demonstrated gas predominantly in the posterior aspect of the upper pole of the left kidney with areas of hypodensity suggesting early parenchymal necrosis. In view of these findings, a percutaneous drain was inserted into the posterior aspect of the upper pole with gradual improvement in the patient's clinical condition. Upper tract decompression with ureteric stent insertion or percutaneous nephrostomy was not pursued due to the absence of radiologic evidence of obstructive uropathy. Intra-operative urine cultures sent at the time of drain insertion were positive for pan-sensitive Escherichia coli with antibiotics rationalised to intravenous Ceftriaxone.

Following observation in hospital over the subsequent 32 days and multiple staged percutaneous drainage procedures (Table 3, Fig. 2A, Fig. 2B, Fig. 2C, Fig. 2D), final progress imaging demonstrated significant reduction in renal and perinephric gas, and reduction in parenchymal necrosis after which the patient was discharged home on 14 days of oral Amoxicillin. Triggers for re-imaging included clinical or biochemical evidence of worsening infection such as new fevers, increasing drain output, or a rise in inflammatory markers. A reduction in percutaneous drain output was also a trigger for re-imaging to assess adequacy of local source control prior to drain removal. Outpatient progress imaging at 4 months’ following presentation demonstrated no residual gas, with mild persistent parenchymal hypodensity suggesting resolving EPN (Fig. 2A, Fig. 2B, Fig. 2C, Fig. 2D). After multidisciplinary discussion between Urology and Radiology departments, the patient has been discharged to the care of his general practitioner.

**Table 3:** – Timeline of radiological progress and insertion/removal of percutaneous drains.

Days after initial presentation	Radiological progress	Intervention
0	Class 3b EPN, retroperitoneal and anterior gas extension	Percutaneous drain insertion into posterior upper pole
	Renal parenchymal hypodensity suggestive of early necrosis	
	Predominantly in posterior upper pole and anterior mid poles	
3	Interval reduction in parenchymal and perinephric gas with interval development of parenchymal fluid collections in the anterior mid pole and posterior upper pole	Percutaneous drain insertion into anterior mid pole and posterior lower pole (Day 5)
8	Interval reduction in parenchymal and perinephric gas	Removal of anterior mid pole (Day 11) and posterior low pole (Day 13)
14	Interval increase in size of anterior mid pole collection	Re-insertion of percutaneous drain into anterior mid pole collection (Day 18)
21	Reduction in size of anterior mid pole and posterior upper pole collections and gas	Progress imaging on Day 25
25	Significant reduction in parenchymal gas and anterior mid pole and posterior upper pole collections	Removal of anterior mid pole and posterior upper pole collections
		De-escalate antibiotics from Ceftriaxone to oral Amoxicillin
32	Minimal gas within posterior upper pole. Stable appearance of parenchymal and perinephric collections compared to Day 25	Discharge from hospital
118	Further reduction in parenchymal and perinephric emphysema. Regions of parenchymal hypodensity suggestive of resolving EPN	Multidisciplinary team consensus for repeat radiologic assessment in 3 months'

**Fig. 2A:**
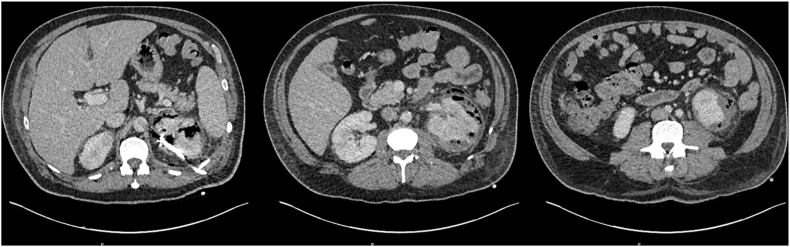
– Progress CTAP Imaging at Day 3 following percutaneous drain insertion into the posterior upper pole (Left to right: upper pole, mid pole, lower pole).

**Fig. 2B:**
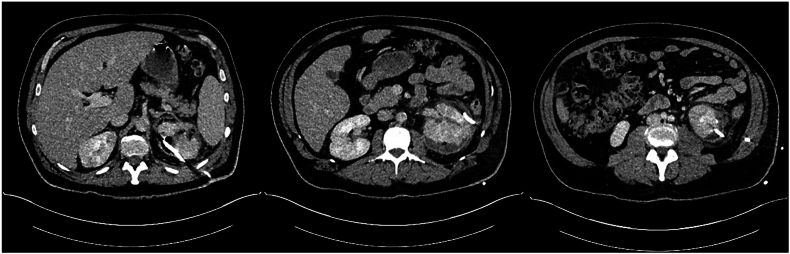
– Progress CTAP on Day 8 following interval insertion of percutaneous drain into anterior mid pole and posterior lower pole with interval reduction in parenchymal gas and interval development of fluid collections.

**Fig. 2C:**
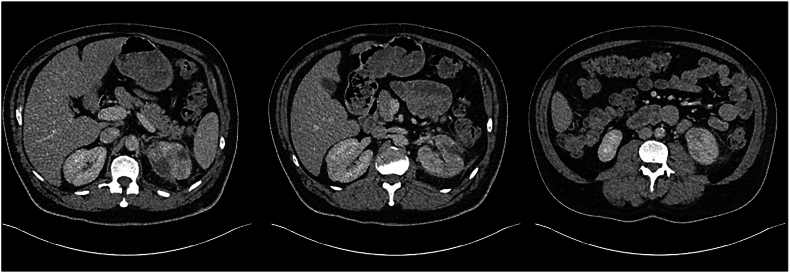
– Progress CTAP on Day 32 following removal of all percutaneous drains demonstrating minimal residual emphysema and residual parenchymal hypodensity suggestive of resolving EPN.

**Fig. 2D:**
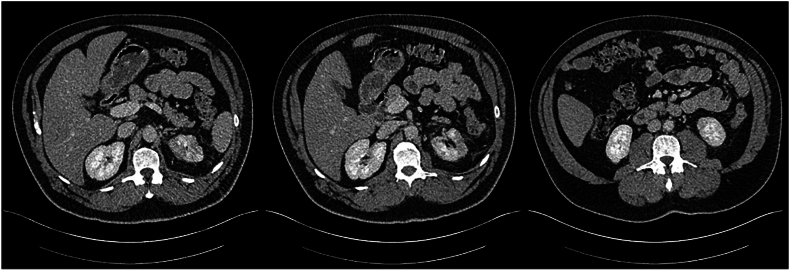
– Month 4 (No residual parenchymal or perinephric gas, resolving emphysematous pyelonephritis).

## 3 Discussion

EPN is a disease associated with significant mortality. Historically, cases managed non-surgically have had reported mortality rates of up to 80 % and the management norm had been open surgical drainage or nephrectomy, particularly in septic patients or those with extensive gas or parenchymal necrosis present on imaging.^3^^,^^7^ Developments in interventional radiology have enabled percutaneous access to the kidney and collecting system, forging the pathway for percutaneous drainage as a minimally-invasive and nephron-sparing alternative to treat EPN.^8^ While less severe cases can be managed conservatively, identifying patients with severe EPN who can be treated effectively with or temporised using percutaneous drainage is more challenging.^3^^,^^7^

In a systematic review by Somani et al., including 210 patients with EPN, the mortality of patients treated with percutaneous drainage was 13.5 % compared with a mortality of 25.0 % in patients treated with emergency nephrectomy. Additionally, of those undergoing percutaneous drainage as the initial method of source control, 13 % required an interval nephrectomy, though, with only a single death in this cohort of patients. Similar outcomes were reported in a single-centre audit of 39 EPN cases by Kapoor et al., who observed a mortality rate of 8 % in patients managed with percutaneous drainage, with no deaths in those requiring subsequent nephrectomy.^9^ Attempts at identifying risk factors for poorer outcomes or failure of non-surgical management have yielded variable results. Clinical features such as altered mental status or septic shock, biochemical parameters such as elevated serum creatinine, thrombocytopenia, or radiological class have been reported as potential predictors of poor outcome by some authors.^7^^,^^9^^,^^10^

To our knowledge, there has been no reported case of emphysematous pyelonephritis that has been managed using multiple staged percutaneous drainage procedures based on frequent re-assessment of radiological disease extent. Historically, cases of extensive EPN have been managed with either upfront nephrectomy, or with percutaneous drainage followed by nephrectomy when there is evidence of patient deterioration^3^. In this case, we demonstrate that adequate source control may be achieved through percutaneous drainage guided by periodic radiologic re-assessment to allow early identification of developing areas of parenchymal necrosis or fluid collections which may be targets for minimally-invasive source control. Triggers for re-imaging included clinical factors such as new fevers or an increase in percutaneous drain output or biochemical factors such as a rise in inflammatory markers. This approach enables adequate source control to be maintained, without awaiting patient deterioration as a trigger for re-assessment or treatment escalation.

Key principles in managing emphysematous pyelonephritis are highlighted in this case including initial resuscitation with intravenous fluids and broad-spectrum antibiotics with coverage of gram-negative uroenteric pathogens, strict glycaemic control, maintaining an unobstructed urinary tract, and finally source control. Initial antibiotic therapy should cover the typical organisms causing EPN and may include beta-lactamase inhibitors, carbapenems, third or fourth generation cephalosporins, with the addition of aminoglycosides in those without contraindications. Antibiotic therapy can subsequently be rationalised once a culprit pathogen and its antimicrobial susceptibilities are identified.^11^ Where there is evidence of upper tract obstruction, decompression with ureteric stenting or percutaneous nephrostomy tube insertion is essential.

In this case report, we present a unique case of severe EPN managed successfully using multiple staged percutaneous drainage procedures based on frequent radiological assessment over a period of 32 days. Such an approach has not been reported in the literature. This case highlights the immense potential percutaneous drainage has as a nephron-sparing technique to managing even severe cases of EPN. We demonstrate that percutaneous drainage is safe when supplemented by frequent clinical, biochemical, and radiological assessment.

## CRediT authorship contribution statement

**Shravankrishna Ananthapadmanabhan:** Writing – original draft, Conceptualization. **Zoe Williams:** Writing – original draft. **Ramesh Shanmugasundaram:** Writing – review & editing, Conceptualization. **Henry Wang:** Writing – review & editing. **Nicholas Mehan:** Writing – review & editing, Supervision.

## 4 Statement of ethics

Informed patient consent has been obtained for publication of this case report and clinical images.

## Declaration of competing interest

The authors have no conflicts of interest to declare.
